# Development of Loop-Mediated Isothermal Amplification Assay for the Detection of aaic Positive Enteroaggregative *Escherichia coli* (EAEC)

**DOI:** 10.1007/s12010-026-05700-2

**Published:** 2026-04-17

**Authors:** Alazar Amare Amdiyee, Tesfaye Sisay Tessema

**Affiliations:** 1 Biotechnology research center, Addis Ababa University, P.O. Box 1176, Addis Ababa, Ethiopia; 2 Department of Medical Laboratory Sciences, College of Medicine and Health Science, Dilla University, P.O. Box 419, Dilla, Ethiopia

**Keywords:** EAEC, LAMP, PCR, Sensitivity, Specificity, Molecular diagnostic

## Abstract

Enteroaggregative *Escherichia coli* is a significant etiologic agent for diarrheal disease globally. Polymerase Chain Reaction (PCR) used as a major detection method for EAEC strains. However, its routine use in diagnostic laboratories is limited by several factors. In this study, a Loop-Mediated Isothermal Amplification (LAMP) assay was developed as an alternative molecular method for detecting aaic positive EAEC strains. The LAMP reaction was performed by incubating the reaction components including 4 sets of designed primers, isothermal buffer, MgSO4, Bst polymerase, dNTPs, and template DNA in a water bath at 61°c for 1 h. The assay performance was evaluated by using 60 bacterial strains and showed high analytical sensitivity, specificity and efficiency on limited strain panel. The developed LAMP assay could detect up to 0.098pg of DNA/reaction. In contrast, the conventional PCR exhibited a detection limit of 0.98pg/reaction. In pure culture, the developed LAMP assay has a lower detection limit of 80 CFU/mL, which is 10-fold lower than that of conventional PCR (8 × 10² CFU/mL), indicating its higher sensitivity. Additionally, in a spiked feces sample, the lowest detection limit for LAMP was 8.2 × 10^2^ cfu/g stool, but the lowest detection limit for PCR was 8.2 × 10^4^ cfu/g stool. Notably, the LAMP assay shows 100-fold lower detection than conventional PCR. The developed LAMP assay detects *aaic*-positive strains and forms a promising foundation for future multiplex assay development aimed at comprehensive EAEC detection.

## Introduction

 Enteroaggregative *Escherichia coli* is a widespread diarrheagenic *E. coli* (DEC) pathotypes, it is associated with episodes of acute and persistent diarrhea in children and adults worldwide. It accounts a considerable portion of the global diarrheal disease burden, being implicated in both epidemic and endemic cases [1–3]. A meta-analysis of 41 studies shows EAEC as a significant cause of acute diarrheal illness in developing countries [4]. The pathophysiology of these strains is mediated by their interaction with human and animal intestinal cells. The in vitro, vivo, and ex vivo investigations show EAEC can stick to the jejunal, ileal, and colonic epithelium and exhibits a distinctive ‘stacked-brick’ adherence pattern, which creates a robust biofilm within a mucus layer, followed by cytotoxic and proinflammatory effects. The clinical symptoms of EAEC infection comprises watery diarrhea, occasionally with blood and mucus [5–7].

Bacterial culture and biochemical tests are inadequate for reliable identification of EAEC strain as they exhibit similar phenotypic characteristics to normal flora and other pathogenic *E. coli* strains. The EAEC can be identified by HEp-2 cell culture-based method, which detects aggregative adherence (AA) pattern exhibited by the bacteria on epithelial cells [8–10]. However, the implementation of HEp-2 cell assay is limited due to the requirement of cell culture facilities, which available only in reference laboratories and prolonged processing time [11, 12].In contrast, PCR used as a major molecular approach for detecting EAEC strains based on the presence/absence of specific virulence genes, including aaic found in a conserved chromosomal region of EAEC, and plasmid associated virulence gene such as *aggR*, *aatA*, or *aap* [13–15]. However, PCR has certain limitation including intensive sample preparation in order to eliminate amplification inhibitors, difficulty in optimizing reaction condition, need for skilled personnel and reliance on expensive thermal cycler equipment, which limits the applicability in routine laboratories. In contrast, loop-mediated isothermal amplification was introduced as alternative to PCR, offering a simple, rapid, and cost-effective detection of the target pathogen [16, 17].

LAMP is first introduced by Notomi et al. in 2000. In contrast to PCR, LAMP is performed under a constant temperature by using *Bacillus stearothermophilus* (Bst) DNA polymerase, which facilitates auto-cycling strand displacement DNA synthesis. LAMP employs a set of four to six specifically designed primers, thereby significantly enhance both the sensitivity and specificity of the assay [18–20].

LAMP requires affordable equipment, such as a water bath or heat block that maintains a constant temperature, making it readily adaptable for use in routine laboratories. Recently, LAMP has been effectively used in detection of various pathogens, including *Salmonella Typhi*,* COVID-19*, *Ebola virus*, *Staphylococcus aureus* and Malaria parasites [20–25]. As EAEC is a common bacterial cause of diarrhea, the development of LAMP method is essential for its quick, inexpensive, and easy detection.

## Materials and Methods

### Bacterial Strains

In the present study the LAMP assay was developed, optimized, and evaluated by using 60 bacterial strains, including 10 Enteroaggregative *Escherichia coli* (EAEC), 10 Enterotoxigenic *Escherichia coli* (ETEC), 10 Enteropathogenic *Escherichia coli* (EPEC), 10 Shiga toxin-producing *Escherichia coli* (STEC), 2 Enteroinvasive *Escherichia coli* (EIEC), and 4 Enterohemorrhagic *Escherichia coli* (EHEC), 10 non-pathogenic *E. coli* strains, and 4 non-*E. coli* bacterial species (*Staphylococcus aureus*,* Pseudomonas aeruginosa*,* Salmonella typhi*,* and Klebsiella pneumoniae*) that were available in glycerol stocks preserved in −80 freezer (Biobase, China) at biotechnology research center, Addis Ababa University. *E. coli* strains were cultured on eosin methylene blue (EMB) agar, while non-*E. coli* bacteria were cultured on nutrient agar at 37 °C for 24 h, then the isolates were sub cultured in 10 ml of TSB at 37 °C for 24 h.

### DNA Extraction

One ml of each bacterial culture grown in TSB was transferred into 1.5 ml sterile Eppendorf tube and centrifuged at 15,000 rpm for 10 min. The supernatant was discarded and the resulting pellet was resuspended in 200 µL of nuclease free water. A centrifugation at 15,000 rpm for 10 min was followed. Upon discarding the supernatant, 100 µL of TE buffer was added to the pellet and incubated at 100C^0^ for 10 min.The sample was then centrifuged at 10, 000 rpm for 2 min and cooled on ice for 1 min. The resulting supernatant was transferred to new sterile Eppendorf tube and stored at −20C^0^ for uses as the DNA template in LAMP and PCR assays. Bacterial DNA extraction kit was used for gram positive bacteria [26, 27]. The concentration and purity of the extracted DNA were measured using the Nanodrop 2000/2000 C Spectrophotometer (Thermo Scientific TM (Thermo Fisher Scientific Inc.), Waltham, MA, USA).

### PCR Amplification

By utilizing specific PCR primers, the conventional PCR assay was run concurrently with the LAMP to verify the results provided in Table 1. The final reaction volume for each PCR experiment was 25 µl and contained 2.5 µL of PCR buffer, 2.5 µL of MgSO4, 0.5 µL of dNTP, 0.5 µL of each forward and reverse primer, 8U of Taq DNA polymerase (Solis BioDyne OÜ, Tartu, Estonia), 15 µL of nuclease-free water, and 3 µL of template DNA. As a negative control, the identical reaction mixture excluding template DNA was utilized. The amplification process involved 35 cycles of 30 s of denaturation, 30 s of annealing, and extension of 1 min, with an initial denaturation temperature of 94 °C for 5 min. A final extension step at 72 °C for 10 min was included [28]. A Prima 96 plus thermal cycler was used for amplification (HiMedia Laboratories Private Limited, Mumbai, Maharashtra, India). The PCR product was subjected to 1.5% agarose gel electrophoresis to analyze DNA fragments in 1X TAE buffer for 1 h at 100 V. The product size was estimated using a 100-bp DNA ladder. A UV gel documentation was used to observe and record the resultant DNA bands [29].

**Table 1:** Nucleotide sequences and amplification features of PCR primers

Primer name	sequence 5` − 3`	Anneling temprature.	Product length	Ref
Forward	ATTGTCCTCAGGCATTTCAC	56 °C	215	[13]
Backward	ACGACACCCCTGATAAACAA			

### LAMP Optimization and Detection of Amplification Products

To determine the LAMP assay’s optimum reaction parameters, a single-factor optimization approach was employed based on the following initial reaction setup: a 25 µL reaction mixture 2.5 µL of Isothermal Amplification Buffer, 1 µL of Bst DNA polymerase (8 U New England Biolabs, Inc., Ipswich, MA, USA), 10.0 mM MgSO₄, 1.2 mM dNTPs, 1.6 µM of FIP and BIP primers, 0.4 µM of F3 and B3 primers, 10 µL nuclease free water and 2.0 µL of template DNA. A reaction mixture lacking of template DNA served as a negative control. The entire mixture was heated to 80 °C for five minutes after being incubated at 65 °C for 60 min [20, 30]. Five essential reaction components including MgSO₄ concentration, reaction temperature, incubation time, dNTP concentration, and Bst DNA polymerase concentration were individually varied in order to further optimize the initial reaction combination. The effects of each of the five factors on amplification efficiency were evaluated at various levels.

In this study LAMP product was confirmed by three different of detection methods, including visual turbidity assessment, SYBR Green I, and gel electrophoresis. LAMP Product detection by SYBR Green I was performed by adding 1µL of SYBR Green I to the amplification product, when amplified DNA products were present, the SYBR green appeared green, but in the absence of DNA amplification, the color stayed orange to the naked eye. The electrophoresis-based detection was performed by adding 3 µL of reaction product to 1.5% agarose for the gel electrophoresis at 100 V for one hour in 1X TAE buffer. A ladder like DNA bands was seen and recorded using a UV transilluminator gel documentation device when target amplification was present. Visual turbidity assessment was another method used to confirm the amplification product.

### Sensitivity, Specificity and Detection Limit of the LAMP Assay

The diagnostic performance of the developed LAMP assay was evaluated by using 60 bacterial isolates, including 10 Enteroaggregative *Escherichia coli* (EAEC) strains and 50 non-EAEC bacterial isolates. Although the number of isolates included in this study is limited due to sample availability, the panel included have a variety of target and non-target isolates, allowing for the assessment of the assay’s analytical sensitivity and specificity. Specificity was defined as the percentage of non-EAEC strains correctly classified as negative, whilst sensitivity was defined as the percentage of EAEC strains correctly identified as positive. Overnight broth cultures of the *E. coli* strain were serially diluted from 10^− 1^ to 10^− 10^ in order to determine the lower detection limit of the developed LAMP assay. Briefly, a single colony of each strain was individually inoculated into 10 ml of TSB and incubated for 24 h at 37 °C. The broth cultures were serially diluted ten times in normal saline. One ml of each dilution was used to prepare the DNA template, and another one ml was plated on plate count agar for the CFU count to assess the assay’s detection limit in terms of the lowest number of cells that could be detected. The limit of detection (LOD) was determined using serial ten-fold dilutions of the extracted DNA, whose initial concentration was measured by the NanoDrop 2000/2000 C Spectrophotometer and expressed in ng/µL. Each dilution was tested under the same PCR and LAMP conditions, and the LOD was defined as the lowest DNA concentration (pg/reaction) that consistently produced a detectable amplification signal. For the LAMP and PCR assays, two microliters of template DNA from each dilution were utilized. Finally, the assay’s lower detection limit was displayed in terms of fewer CFU/ml and DNA concentration/reaction.

### LAMP Application on Human Fecal Samples Spiked With EAEC

From Ten independent human fecal sample obtained from a healthy donor and from each one g was taken and diluted was diluted in 9 ml of phosphate-buffered saline (PBS). Samples were vortexed for 10 s, allowed to settle for 2 min, vortexed again; then, 1 ml of stool suspension was added to 10 separate tubes and spiked with 1 ml of serially diluted (10^− 1^ to 10^− 10^) of EAEC bacterial suspension. Afterwards 1 ml of each spiked fecal sample dilution was transferred to an Eppendorf tube containing 10X EDTA. Samples were vortexed for 30 s and then centrifuged at 15,000 rpm for 15 min; the supernatants were discarded, and 300 µl of nuclease free water was added; then centrifuged again for 15,000 rpm for 10 min. The supernatants were discarded; then the pellets were suspended in 100 µl 1x TE buffer and boiled at 100 °C for 15 min. After boiling centrifugation was performed at 15,000 rpm for 5 min. Then the supernatant, to be used as template DNA was transferred to a new sterile Eppendorf tube; then the template subjected to PCR and LAMP assay. Due to limited access and availability, clinical stool specimens were not included; instead, spiked stool samples were utilized to assess the assay’s analytical performance. As a negative control, un spiked feces were used.

### Data Analysis

MedCalc software version 23.4.5 was used to calculate and assess diagnostic performance parameters, including sensitivity, specificity, positive predictive value (PPV), negative predictive value (NPV), and accuracy, using a 2 × 2 contingency table. Cohen’s kappa (κ) statistic was also used to evaluate the degree of agreement between the PCR and LAMP assay results.

## Results

### Primer Design for LAMP Assay

A set of four lamp primers were designed by targeting aaic gene (Gene bank accession no:> NC_017626.1(4895043–4895549)) by using the following software: https//www.optigene.co.uk/custom services lamp designer software, http:lamp.neb.com . An aaic gene is located on the bacterial chromosome and is usually more stable than plasmid-associated virulence genes. However, EAEC is a pathotype that varies in its virulence gene composition. Therefore, the designed primer for LAMP assay can identify strains of EAEC that harbor aaic-genes, but it cannot identify strains that are aaic-negative. Therefore, the study’s findings support the incorporation of aaic to a multiplex LAMP technique for EAEC detection. Combining aaic with other EAEC-associated markers, would increase diagnostic accuracy and cover more EAEC subgroups. The designed primer for LAMP assay includes inner primers (Forward inner primer-FIP and Backward inner primer-BIP) and outer primers (Forward outer primer-F3 and Backward outer primer-B3). The overall information of primer sequences is provided in (Table 2).

**Table 2:** Information of designed LAMP primer sequences

Target	Name	Sequence 5` − 3`	Length (bp)	Ref.
aaic	Forward inner primer (FIP)	ACCTGATTTAGTTGATTCCCTACGTCTGGAGAACTTTTTTAAGAGAGG	48	This study
	Backward inner primer (BIP)	TCATTTAAGGTTGTTTATCAGGGGTGAATCATTATCATGAGCCAACA	47	
	Forward outer primer (F3)	GGTTACTAAACACATACAAGACC	23	
	Backward outer primer (B3)	ACGACAATTCCAGAATGAGAT	21	

### Optimization Process of LAMP Assay

**Fig. 1:**
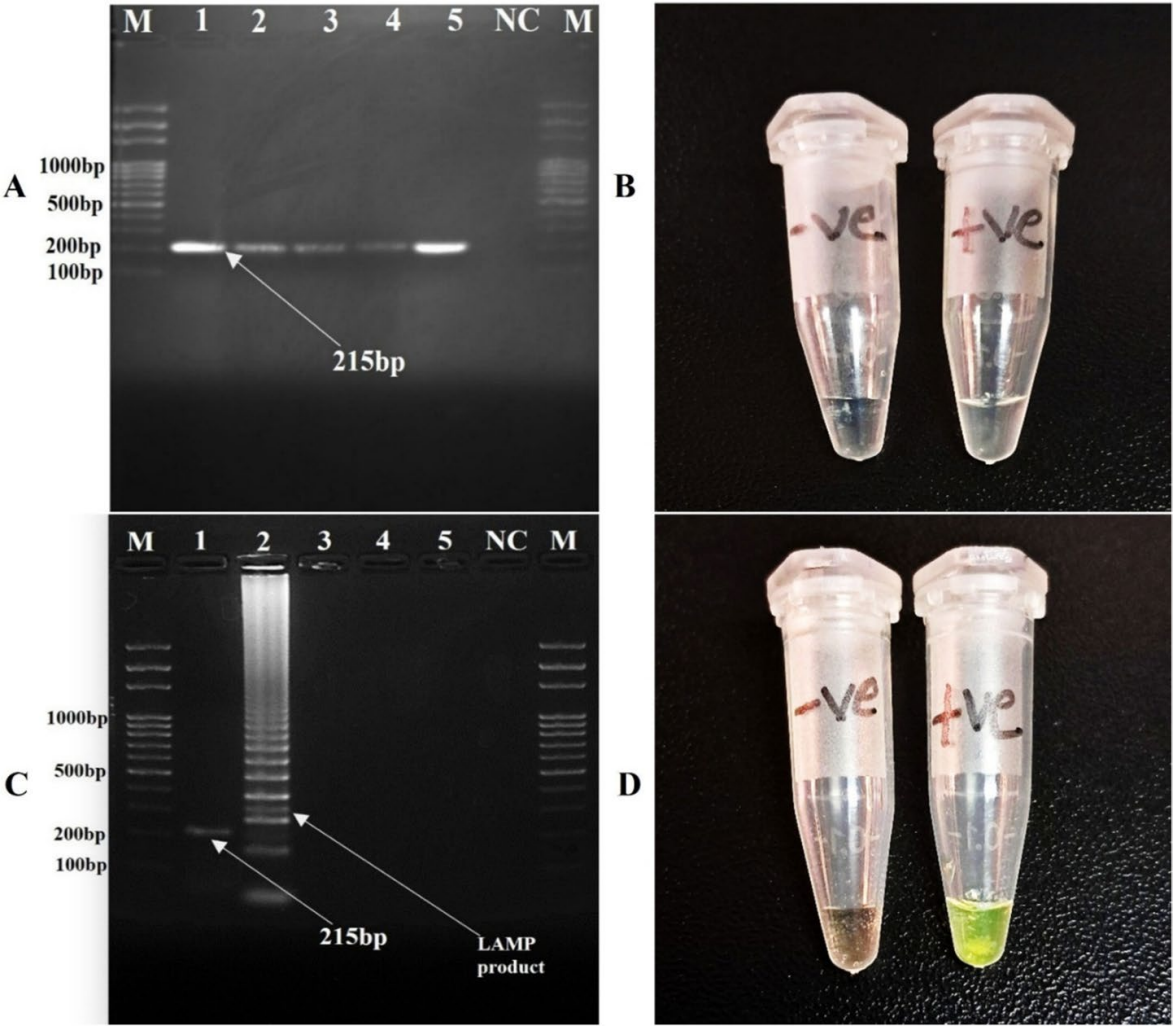
**A**) PCR and LAMP based Detection of EAEC. (**A**) Shows agarose gel electrophoresis PCR product. (**B**) visual turbidity assessment of LAMP Product (**C**) Agarose gel electrophoresis of LAMP product (**D**) SYBR Green I detection of LAMP product

Initial re-confirmation of EAEC strain was carried out by PCR (Fig. 1). The intended lamp primer’s efficacy was then confirmed by using the LAMP assay based on the first reaction condition as outlined in the method section. The amplification result was evaluated by using: Turbidity assessment (Fig. 1b) agarose gel electrophoresis (Fig. 1c) and SYBR Green I dye detection (Fig. 1 d). After five crucial parameters were systematically optimized, the LAMP assay’s optimum reaction condition was determined. The most effective combination of parameters to improve the LAMP assay’s reaction efficiency and specificity was found through this methodical optimization procedure. The detailed optimization process and corresponding results for each factor are presented below.

#### Optimization of MgS04 Concentration

**Fig. 2:**
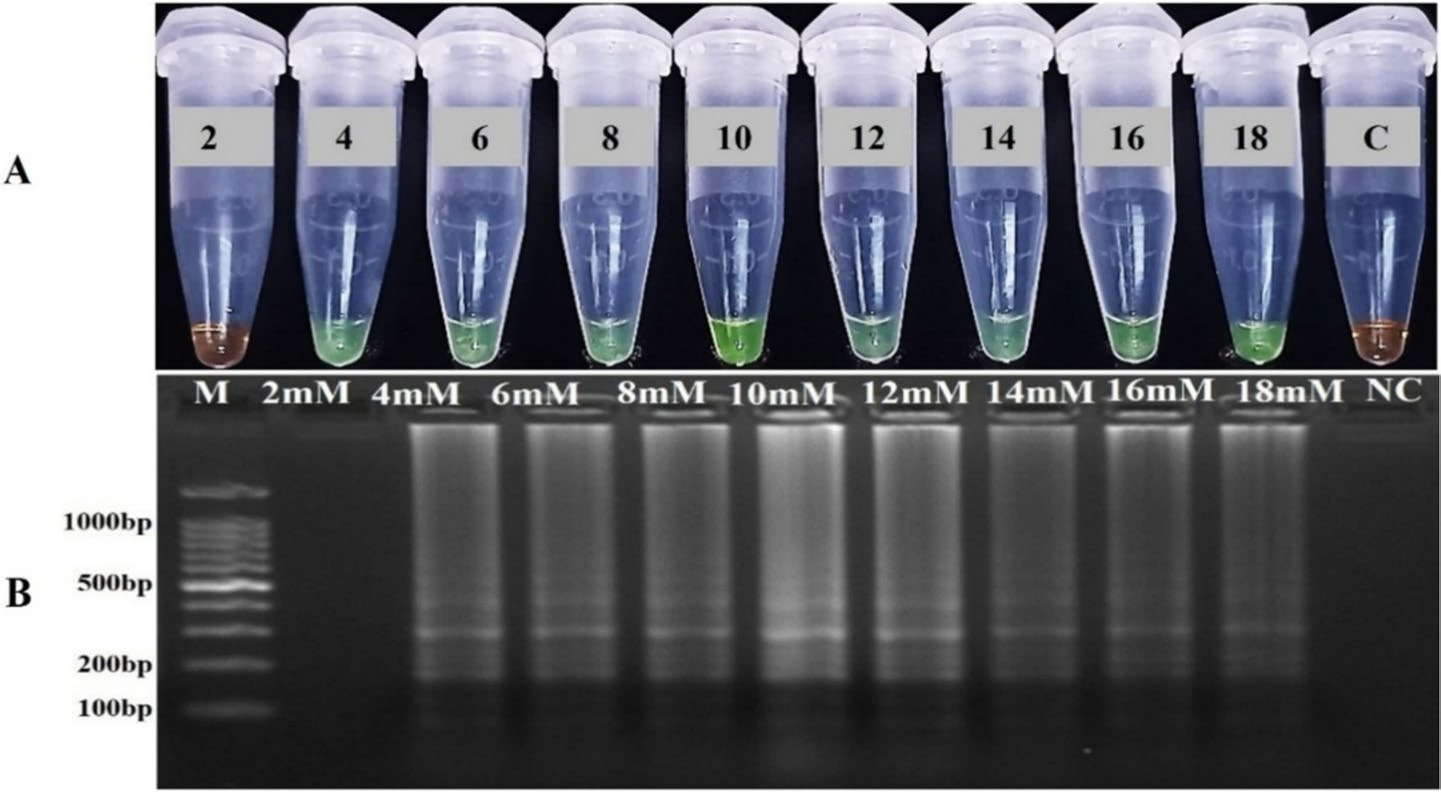
LAMP reactions performed with varying MgSO₄ concentrations ranging from 2.0 to 18 mM (**A**). SYBR Green I visualization (**B**). Agarose gel electrophoresis result

The optimization of MgSO4 concentration was performed by testing MgSO4 ranged from 2.0 to 18 mM concentration and the result showed a green color in a range from 4.0 to 18.0 mM after adding of SYBR green I. The green color in SYBR Green I staining indicates the presences of amplification at tested concentration and the appearance of orange color at 2.0 mM concentration indicates the absence of amplification (Fig. 2a). In agarose gel electrophoresis, the LAMP products displayed as a ladder-like bands from the reaction with the Mgso_4_ concentrations ranging from 4.0 to 18.0 mM. The robust band was appeared at 10.0 mM. Therefore,10.0 mM MgSO4 concentration for the reaction is considered as an optimal MgS04 Concentration (Fig. 2b).

#### Optimization of Temperature

**Fig. 3:**
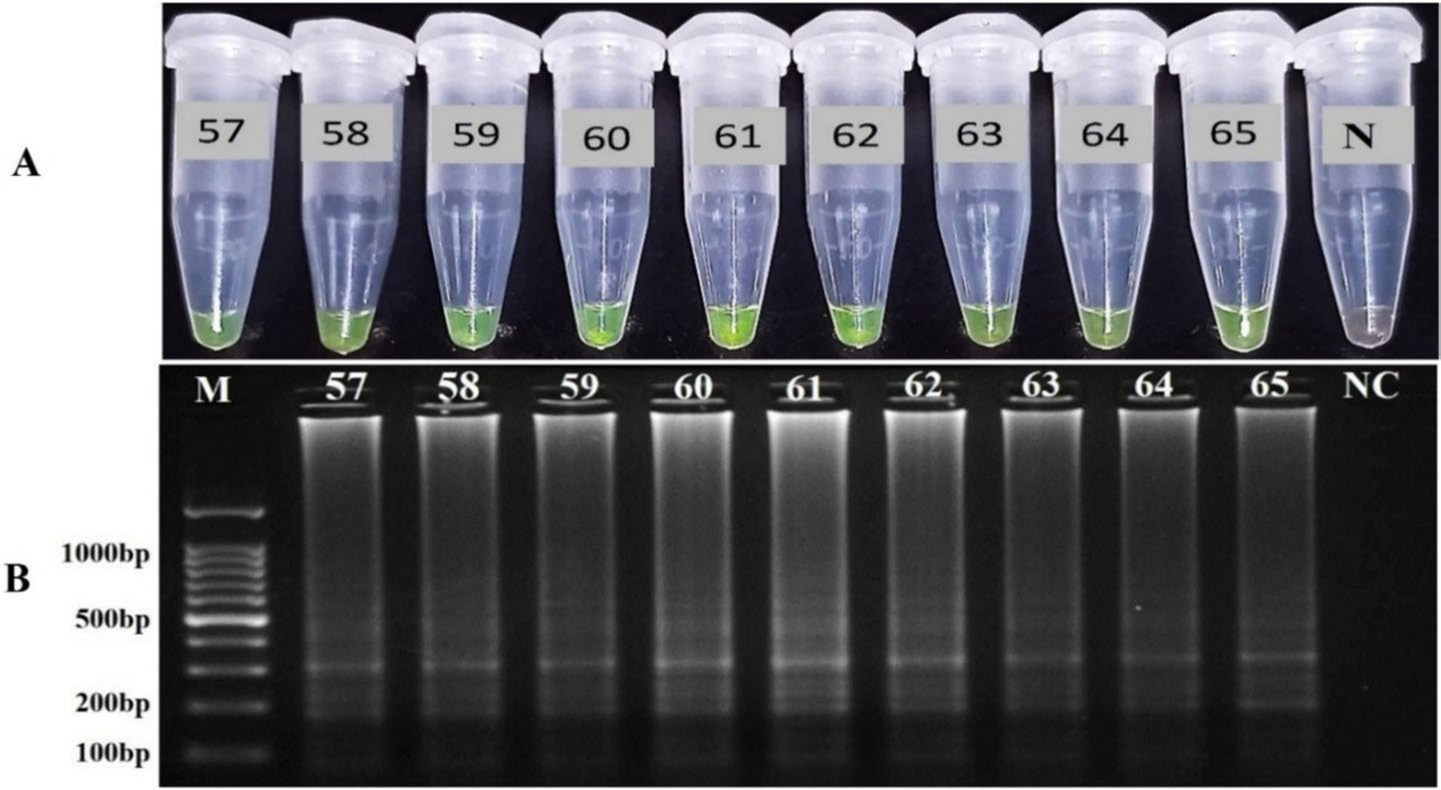
Results of the LAMP reactions tested at a temperature of 57,58,59,60,61,62,63,64,65 respectively. (**A**) SYBR green I detection. (**B**) Agarose gel electrophoresis results

Different temperature ranging from 57 °C to 65 °C was evaluated, a fluorescent green color was displayed at all temperature ranges after SYBR Green I staining (Fig. 3a) and in the agarose gel a ladder-like bands were observed in all evaluated reaction temperatures. The brightest band was observed at 61 °C (Fig. 3b). Therefore, 61 °C considered as an optimal temperature for the LAMP reaction.

#### Optimization Incubation Time

**Fig. 4:**
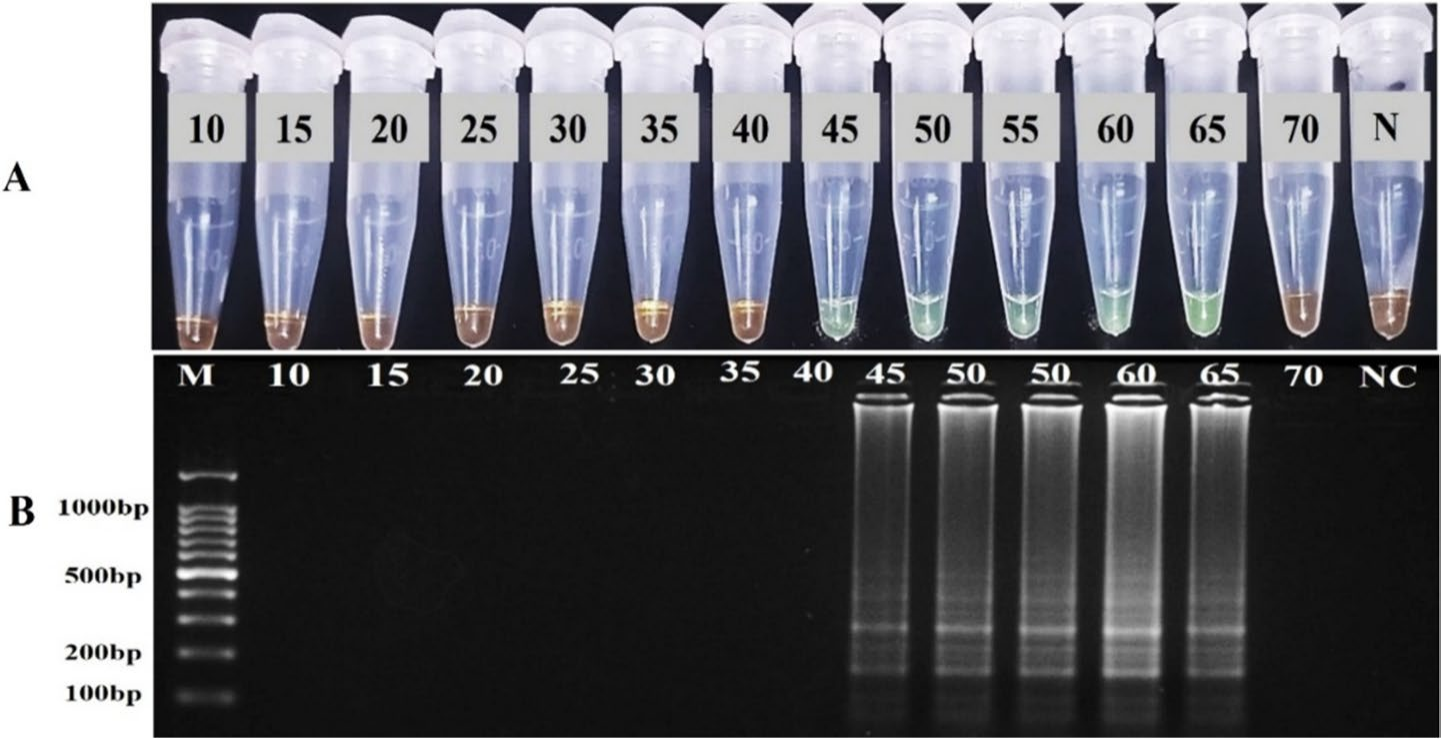
Profiles of LAMP reactions at different incubation times. (**A**) SYBR Green I visualization. (**B**) Gel electrophoresis; tubes/lane 1–13 represents reaction times of 10, 15, 20, 25, 30, 35, 40, 45, 50, 55, 60, 65, and 70 min, respectively; tube 14 represents the negative control

To determine optimal time for LAMP reaction the incubation time ranging from 10 up to 70 min was evaluated. No color change was observed by SYBR green I staining until 40 min, as the color remained orange, starting from 45 up to 65 min a fluorescent green color was displayed indicating the positive result and at 70 min no color change was observed (Fig. 4a). In agarose gel electrophoresis, the LAMP products displayed ladder-like bands starting from 40 up to 65 min. No ladder-like bands were seen between 10 up to 40 and 70 min. The robust band was produced at 60 min.Therefore, the 60 min incubation time for LAMP reaction was considered as an optimal incubation time for the LAMP reaction (Fig. 4b).

#### Optimization of Bst Polymerase Concentration

**Fig. 5:**
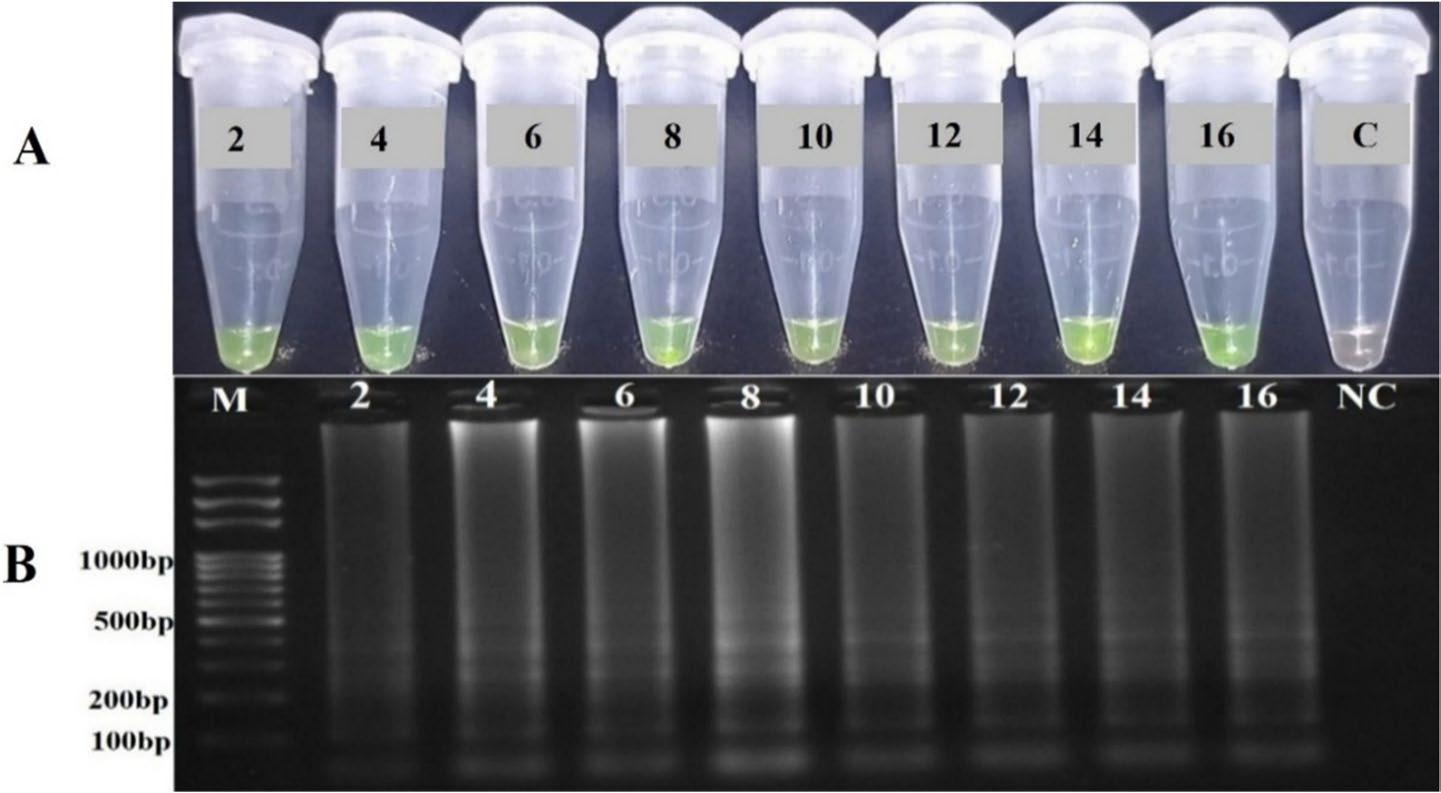
Results of the LAMP reactions using different Bst DNA Polymerase concentrations (2.0, 4.0, 6.0, 8.0, 10.0, 12.0, 14.0, and 16.0 U, respectively). **A**) SYBR Green I visualization. (**B**) Agarose gel electrophoresis result

The bst polymerase concentration ranging from 2-16U were evaluated and a fluorescent green color was displayed in all evaluated concentration after SYBR Green I staining, indicating the presence of amplification (Fig. 5a). The robust band was displayed at 8.0 U of bst DNA polymerase. Therefore, 8U bst polymerase considered as optimal concentration for the reaction (Fig. 5b).

#### Optimization of DNTPs Concentration

**Fig. 6:**
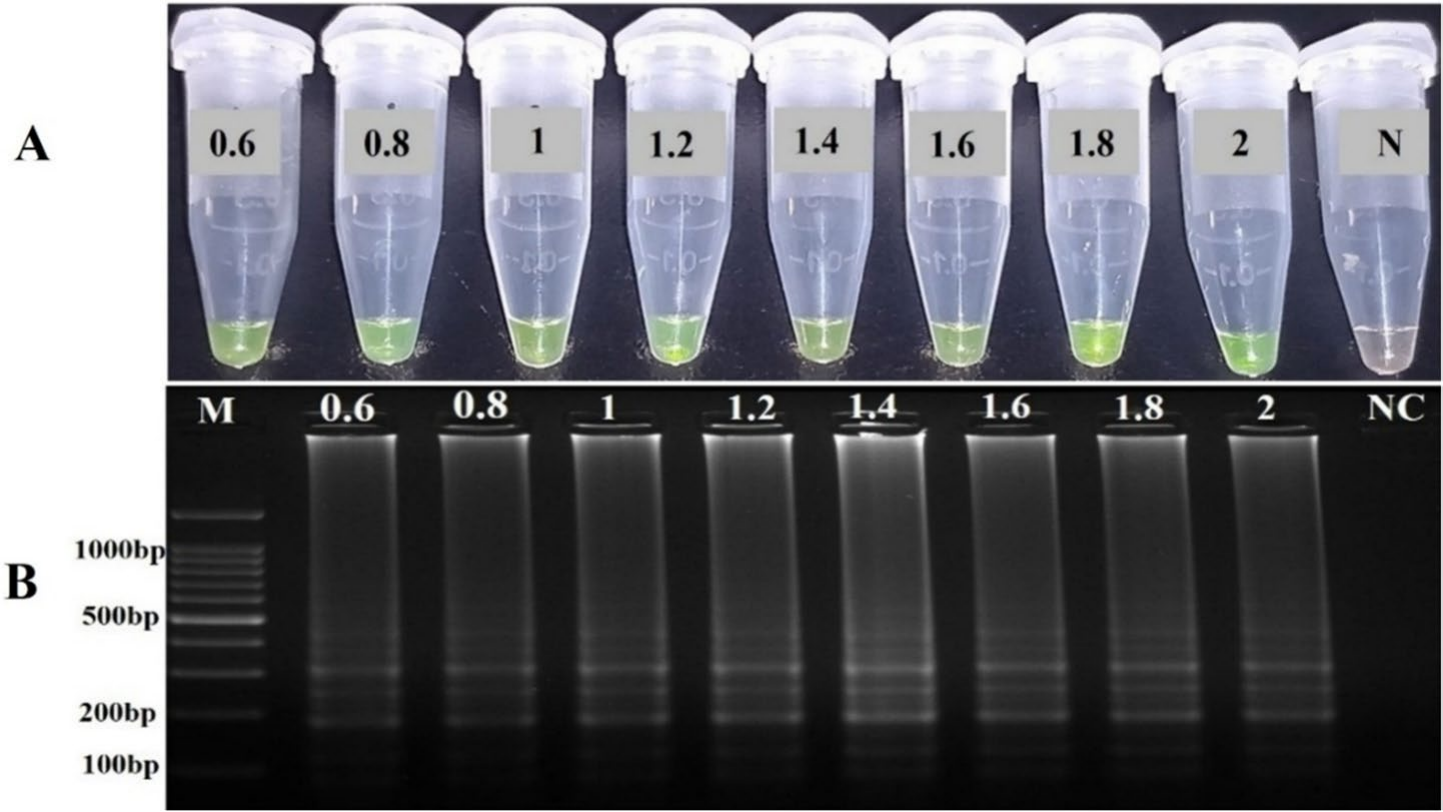
Different dNTP concentrations were tested at 0.6, 0.8, 1.0, 1.2, 1.4, 1.6, 1.8, and 2.0 mM, respectively. (**A**) Shows SYBR Green I visualization. (**B**) Shows agarose gel electrophoresis result

All nine different concentration of dNTPs ranging from 0.6 to 2 mM showed a fluorescent green color in SYBR Green I staining, indicating the target DNA amplified with dNTP concentrations ranging between 0.6 and 2 mM (Fig. 6a). Agarose gel electrophoresis result showed a ladder-like bands in all evaluated dNTPs concentration, LAMP products showed a robust band in reactions with 1.4mM dNTPs. Therefore, 1.4mM considered as an optimal dNTP’s concentration for the LAMP reaction (Fig. 6b).

#### Application of Optimized LAMP Protocol

**Fig. 7:**
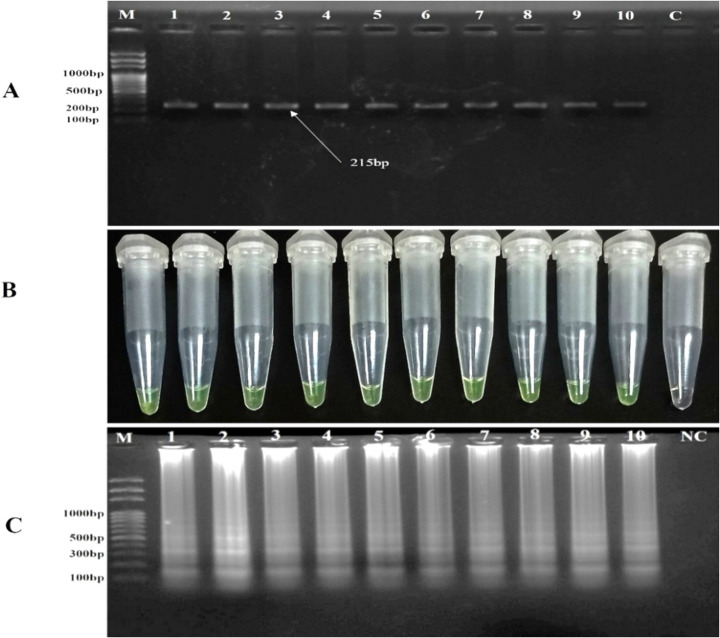
SYBR green I detection of Lamp product of EAEC amplification. **B**) Gel electrophoresis detection of LAMP product. Lane M: DNA ladder; Lanes/Tubes 1–10: EAEC positive Samples; Lanes/Tubes 11: negative control

The optimum reaction conditions were established after evaluations of five critical reaction factors, the final lamp reaction was carried out in a total of 25 µL volume containing 2.5 µL of isothermal amplification buffer, 10 mM of MgSO4 solution, 1.4 mM of dNTPs, 1.2 mM each of FIP and BIP, 0.4 mM each of F3 and B3, 8.0 U of Bst polymerase and 2.0 µL of the target DNA template and the reaction mixture was incubated at 61 C^0^ for 60 min. This optimized condition was used for subsequent LAMP reaction on ten EAEC strain. The result of LAMP amplification was verified by SYBR Green I staining color change, and agarose gel electrophoresis, which revealed the characteristic of ladder-like bands of LAMP products (Fig. 7).

### Validation of LAMP Assay

60 bacterial isolates were used for the LAMP assay, and PCR was carried out concurrently to confirm the results (Table 3). The tested strains were classified as true negative when they were PCR-negative and as true positive when they were PCR-positive. On the other hand, test negative indicates that the strains were negative by LAMP assay and test positive indicates that the strains was positive by LAMP assay. The LAMP assay’s sensitivity, specificity, and overall performance were evaluated using a 2 × 2 contingency matrix (Table 4).

**Table 3:** Parallel comparison of LAMP and PCR

Isolate code	Bacterial strain	PCR assay	LAMP assay
024	EAEC	**+**	**+**
61 C	EAEC	**+**	**+**
72I	EAEC	**+**	**+**
7I	EAEC	**+**	**+**
73I	EAEC	**+**	**+**
72E	EAEC	**+**	**+**
82 F	EAEC	**+**	**+**
EV10.	EAEC	**+**	**+**
EV71	EAEC	**+**	**+**
IH 64	EAEC	**+**	**+**
46 A	EPEC	-	-
C89	EPEC	-	-
C75	EPEC	-	-
C71	EPEC	-	-
C51	EPEC	-	-
CF31	EPEC	-	-
EV96	EPEC	-	-
IH 31	EPEC	-	-
CF28	EPEC	-	-
IH 10	EPEC	-	-
CF10	ETEC	-	-
73 A	ETEC	-	-
C105	ETEC	-	-
C106	ETEC	-	-
C114	ETEC	-	-
C93	ETEC	-	-
C104	ETEC	-	-
C99	ETEC	-	-
C102	ETEC	-	-
C95	ETEC	-	-
C19	EHEC	-	-
C39	EHEC	-	-
C141	EHEC	-	-
C46	EHEC	-	-
EV95	STEC	-	-
IH 85	STEC	-	-
IH 86	STEC	-	-
S04	STEC	-	-
C18	STEC	-	-
C12	STEC	-	-
IH 12	STEC	-	-
C35	STEC	-	-
CF 49	STEC	-	-
S04	STEC	-	-
C57	EIEC	-	-
C30	EIEC	-	-
C63	Non-pathogenic *E. coli*	-	-
C68	Non-pathogenic *E. coli*	-	-
C38	Non-pathogenic *E. coli*	-	-
C40	Non-pathogenic *E. coli*	-	-
C126	Non-pathogenic *E. coli*	-	-
C82	Non-pathogenic *E. coli*	-	-
C 137	Non-pathogenic *E. coli*	-	-
C142	Non-pathogenic *E. coli*	-	-
C143	Non-pathogenic *E. coli*	-	-
CF 45	Non-pathogenic *E. coli*	-	-
P1	*Pseudomonas aeruginosa*	-	-
K3	*Klebsiella pneumonia*	-	-
S2	*Staphylococcus aureus*	-	-
Sal	*Salmonella typhi*	-	-

**Table 4:** 2x2 Contingency matrix table generated by MedCalc Version 23.2.1 software

Test	Present	N*o*	Absent	N*o*	Total
Positive	TP	10	FP	0	10
Negative	FN	0	TN	50	50
Total	-	10		50	60

#### Specificity of the LAMP Assay

**Fig. 8:**
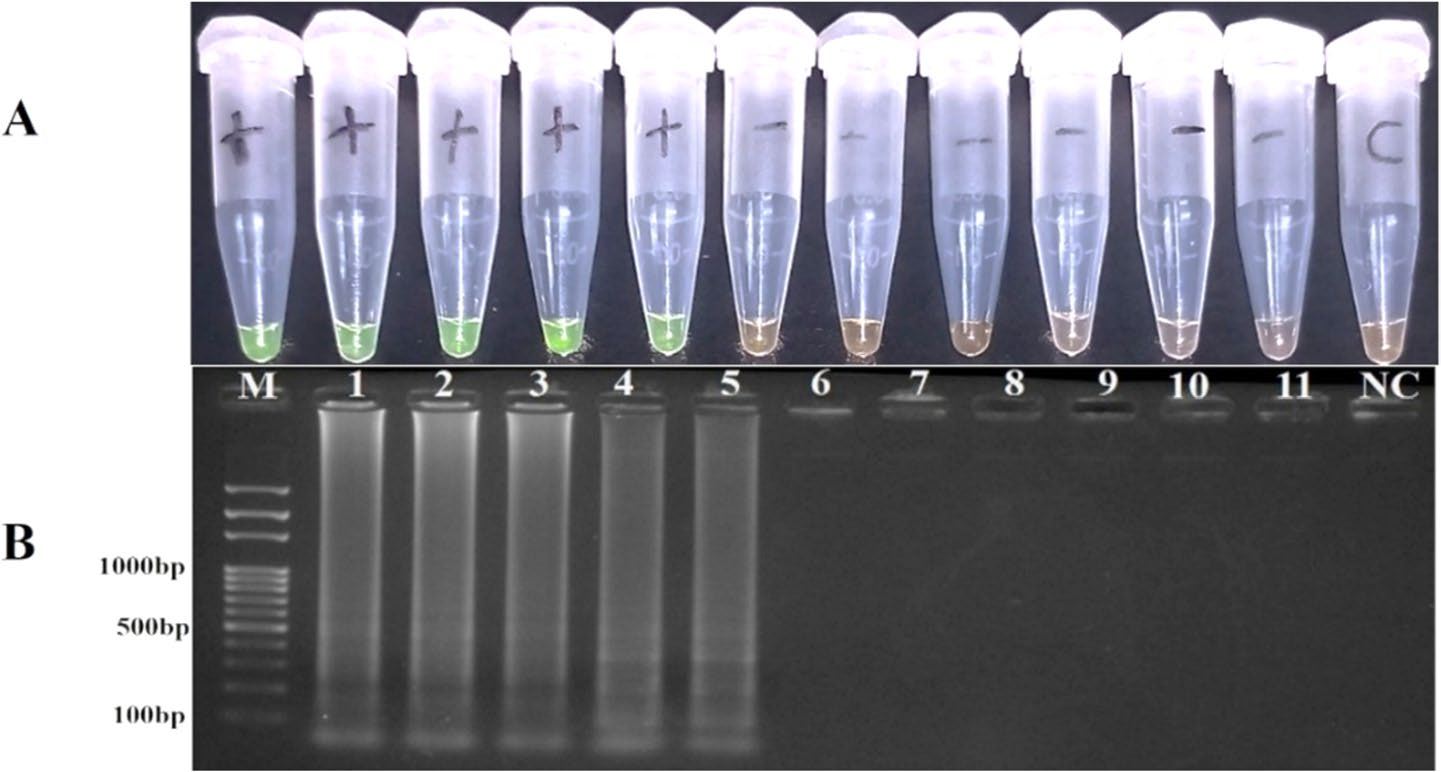
Specificity evaluation of the LAMP assay against non-E. coli bacterial strains. (**A**) Detection using SYBR Green I. (**B**) Agarose gel electrophoresis results: Lane M, 100 bp plus DNA ladder; Lanes/Tubes 1–5, EAEC positive controls; Lanes/Tubes 6–11, non-EAEC *E. coli* strains; Lane/Tube 12, negative control

**Fig. 9:**
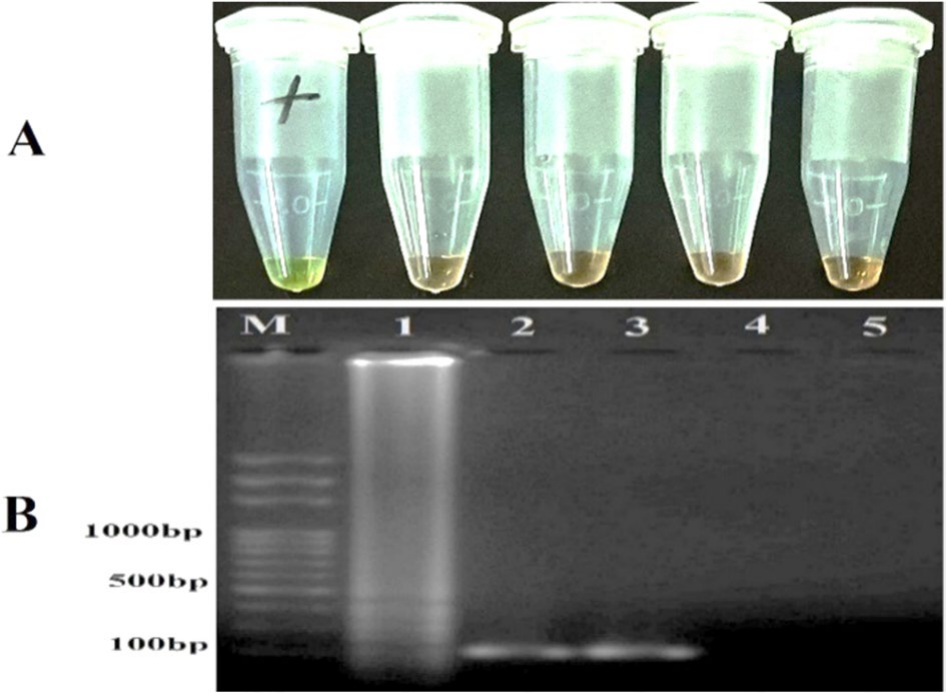
Evaluation of assay specificity against non-*E. coli* bacterial species: (**A**) SYBR green I detection;** B**) Gel electrophoresis result; lane M: DNA ladder (100plus); Lane/Tube 1: EAEC positive control; Lane//tube 2–5 non-*E. coli* bacterial strain

The specificity of the developed LAMP assay was assessed by using 60 bacterial isolates containing 10 EAEC, 46 non-EAEC *Escherichia coli* strains and 4 non-*E. coli* bacterial species. As shown in (Fig. 8) none of the non-EAEC *E. coli* isolates showed any positive results. Furthermore, the assay’s genus-level analytical specificity and absence of cross-reactivity were confirmed by the lack of amplification in the non-*E. coli* species (Fig. 9). The LAMP assay yielded negative result for all 50 non -EAEC bacterial, indicates absence of false-positive results confirms the assay’s high analytical specificity on limited sets of tested isolates (Table 5). Since EAEC is a genetically diverse pathotype, not all strains may carry the aaic gene. As a result, even if the assay demonstrates high analytical specificity, it might not be able to identify all EAEC variant. However, this assay offers a basis for further development of multiplex LAMP assay that incorporate more EAEC virulence markers to increase overall EAEC detection coverage.

**Table 5:** Statistical analysis to assess the LAMP assay’s diagnostic performance Using MedCalc Version 23.2.1 Software for EAEC

Statistic	Formula	Value	95%CI
Sensitivity	(TP/(TP + FN))100%	100.000%	69.150% to 100.000%
Specificity	(TN/(TN + FP))100%	100.000%	92.888% to 100.000%
Positive predictive value	(TP/(TP + FP))100%	100.000%	69.150% to 100.000%
Negative predictive value	(TN/(TN + FN))100%	100.000%	92.888% to 100.000%
Accuracy	(TP + TN)/(TP + TN +FP + FN) 100%	100.000%	94.037% to 100.000%

#### Sensitivity of the LAMP Assay

The developed LAMP assay detected all 10 EAEC tested isolates under the evaluated conditions. However, further validation using a larger number of true clinical samples and geographically diverse EAEC is required to assess clinical sensitivity and overall assay’s performance. Furthermore, the study demonstrated a positive predictive value of 100%, indicating that every sample tested positive by the LAMP assay was identified as an EAEC isolate. Concurrently, a negative predictive value of 100% was noted, indicating that every negative result accurately determined absence of aaic-positive EAEC. Negative results might not rule out all EAEC variants, due to the existence of strains that lack aaic gene. Therefore, adding more EAEC virulence targets to a multiplex assay could improve detection coverage of EAEC. The findings in this study shows high degree of agreement between the LAMP assay results on all tested samples for the assay (Table 5).

#### Cohen’s Kappa Test Statistics

The LAMP and PCR assay’s degree of agreement was evaluated by using Cohen’s Kappa (K). The result showed perfect agreement with a Kappa value of 1.00, which indicates, the LAMP and PCR tests demonstrated perfect agreement across all tested isolates. This outcome demonstrates that the LAMP test and PCR are equally effective in identifying the target strains (Table 6.).

**Table 6:** Kappa test statistics(K)

LAMP	PCR				Weighted Kappa	95%CI
	Test	Positive	Negative	Total	1.0	1.00 to 1.00
	Positive	10	0	10		
	Negative	0	50	50		
	Total	10	50	60		

#### Lowest Detection Limit of LAMP Assay (LOD)

**Fig. 10:**
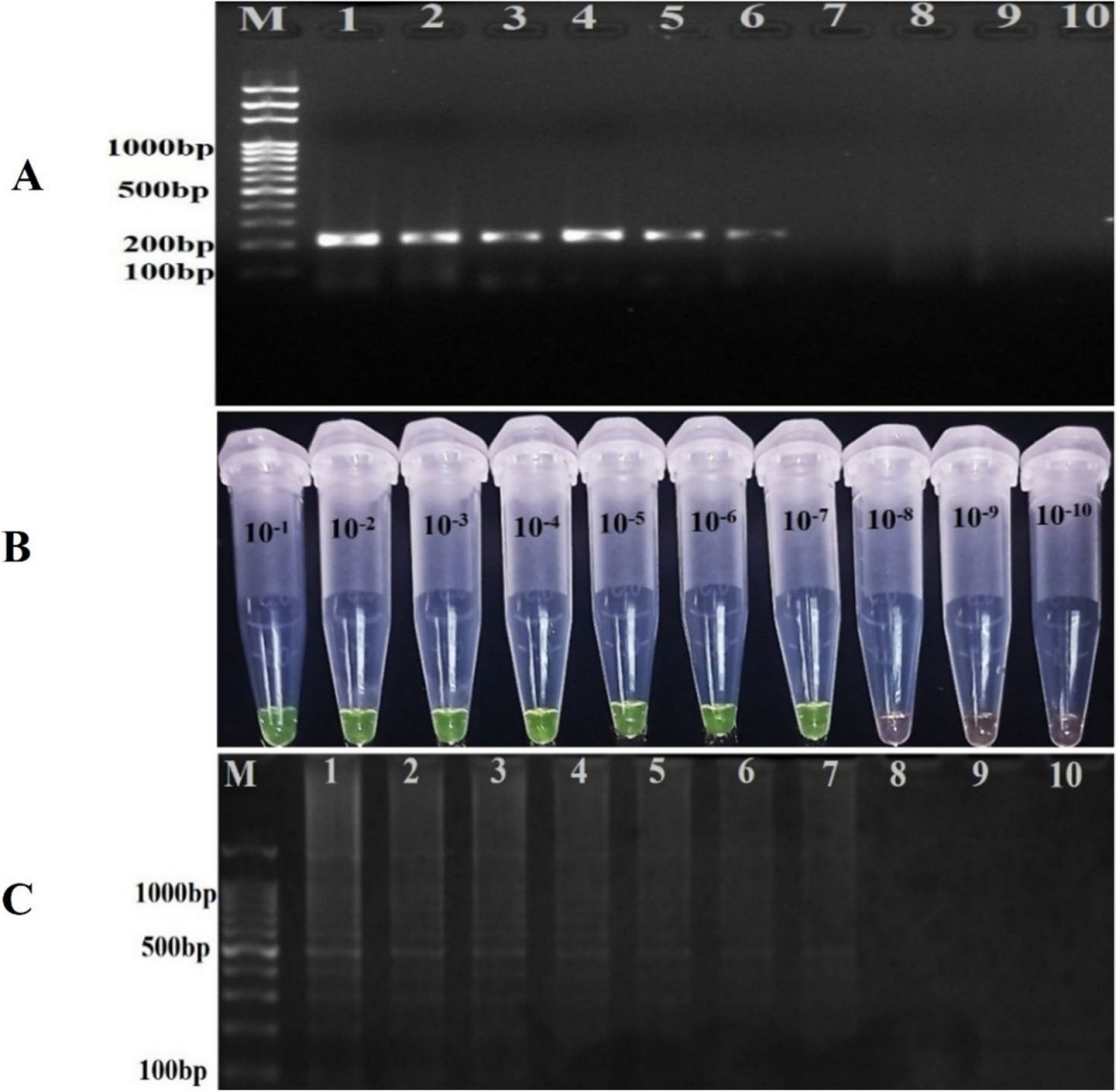
Comparison of the detection limit of PCR and LAMP assays using 10-fold serial dilutions of EAEC DNA. (**A**) PCR products analyzed by gel electrophoresis. (**B**) LAMP products detected by SYBR Green I visual inspection. (**C**) LAMP products analyzed by gel electrophoresis. Lane M: DNA ladder; Lanes/Tubes 1–10: serial dilutions 10⁻¹ to 10⁻¹⁰; NC: negative control

The developed lamp assay detection limit was determined by performing a series of 10-fold serial dilutions of EAEC template DNA. The LAMP assay shown a positive result as low as 0.098pg of DNA template per reaction, indicating the lowest detection limit of the LAMP assay was 0.098pg/reaction. In contrast, the conventional PCR has a lowest detection limit of 0.98 pg/reaction. The result of both LAMP and PCR across the dilution series are presented in (Table 7), and the corresponding amplification results are shown in (Fig. 10). Standard plate counting method was used to determine the numbers of bacterial cells detected by the developed LAMP assay. The total viable count of undiluted culture was 8 × 10^9^CFU/mL by using plate-counting method. The result of LAMP and PCR assay shows a notable difference in detection limits. Specifically, the LAMP assay exhibited 80 CFU/mL lower detection limit and conventional PCR has 8 × 10^2^CFU/ml. This indicates the developed LAMP assay has 10-fold lower detection limit than conventional PCR in identifying the target EAEC in pure culture. Additionally, the lowest detection limit for LAMP in a spiked stool sample was 8.2 × 10^2^ cfu/g stool, but the lowest detection limit for PCR was 8.2 × 10^4^ cfu/g stool, indicating that LAMP has 100x lower detection limit than PCR. The spiked stool samples experiment for evaluating the analytical performance of the LAMP assay demonstrated high analytical sensitivity and specificity. Future studies should include clinical specimens to comprehensively assess the assay’s performance and support its widespread use.

**Table 7:** Summary of serial dilution result, showing a comparative detection limit for both LAMP and PCR

Dilution Factor	DNA concentration	DNA concentration per reaction (2 µL template).	LAMP	PCR
10^− 1^	49.0ng/µL	98*10^1^ ng/reaction	**+**	**+**
10^− 2^	4.90ng/µL	9.80*10^0^ ng/reaction	**+**	**+**
10^− 3^	0.490ng/µL	9.80*10^− 1^ ng/reaction	**+**	**+**
10^− 4^	0.0490ng/µL	9.80*10^− 2^ ng/reaction	**+**	**+**
10^− 5^	4.90 pg/µL	9.8 pg/reaction	**+**	**+**
10^− 6^	0. 490 pg/µL	0.98 pg/reaction	**+**	**+**
10^− 7^	0.0490pg/µL	0.098pg/reaction	**+**	**-**
10^− 8^	0.00490pg/µL	0.0098 pg/reaction	-	-
10^− 9^	0.000490pg/µL	0.00098 pg/reaction	-	-
10^− 10^	0.0000490pg/µL	0.000098 pg/reaction	-	-

## Discussion

Diarrheal disease is a significant cause of death and economic losses in developing countries, it is caused by a diverse group of bacteria, viruses, and parasites. Among bacterial pathogens, diarrheagenic *Escherichia coli (*DEC) are common and classified into; Enteroaggregative *Escherichia coli* (EAEC), Enterohemorrhagic *Escherichia coli* (EHEC), Enteropathogenic *Escherichia coli* (EPEC), enterotoxigenic *Escherichia coli* (ETEC), Enteroinvasive *Escherichia coli* (EIEC), diffusely adherent *Escherichia coli* (DAEC), Shiga toxin producing *Escherichia coli* (STEC), adherent-invasive *Escherichia coli* (AIEC) and cell-detaching *Escherichia coli* (CDEC) strains [31–33]. Among DEC strains, enteroaggregative *Escherichia coli* (EAEC) is a significant pathogen responsible for causing acute and persistent diarrhea globally [34].

The widespread prevalence of diarrheal diseases globally emphasizes the need for rapid, affordable, accurate, and easy diagnostic method for the detection of the causative microorganisms. LAMP is an innovative molecular assay emerged as an alternative to PCR for rapid, accurate, and affordable diagnosis. Several studies that have been conducted with LAMP shows its impressive role in addressing the gap in molecular diagnostics [16, 35, 36]. Recently, LAMP assays have been developed for various diarrheal pathogens including pathogenic *Escherichia coli* strains such as Shiga toxin producing *Escherichia coli*, enterohemorrhagic *Escherichia coli*, enterotoxigenic *Escherichia coli* [37–39]. Furthermore, LAMP has been effectively used for the diagnosis of other diarrheal microorganism including, *clostridioides difficile*, *shigella flexneri*,* Vibrio cholerae*,* Entamoeba histolytica*, *rotavirus* and Giardia *lamblia* [40–45].

In this study we developed loop mediated isothermal amplification assay for detection of Enteroaggregative *Escherichia coli* (EAEC). The target gene for the LAMP primer design for EAEC was the aaic gene. It is found on the bacterial chromosome and is typically more stable than virulence genes linked to plasmids, which can be lost or passed from strain to strain. Conversely, EAEC is a genetically heterogeneous pathotype with a variable makeup of virulence genes. Some study indicates that not all EAEC isolates include aaic. Therefore, targeting only on aaic can detect all EAEC strains seen in clinical or environmental settings [46].

The present study showed high analytical sensitivity and specificity for EAEC detection. The assay reliably identified EAEC strains harbor the aaic gene. In comparison to previous study where the LAMP assay shows 100% sensitivity and 97.05% specificity for detecting enterohemorrhagic *Escherichia coli* (EHEC) [47], the specificity of developed lamp assay is higher. Furthermore, the LAMP and PCR shows a perfect agreement on tested isolates. This level of agreement is higher than previously reported LAMP assays for EHEC detection [47].

The developed LAMP assay was able to detect DNA at concentrations as low as 0.098 pg/reaction, whereas conventional PCR showed a lower detection limit of 0.98 pg/reaction. This indicates the developed LAMP assay has approximately 10 times lower detection limit than PCR. The results are consistent with previous LAMP assay having the detection limit of 0.5-0.5.1pg/reaction for Shiga toxin producing *Escherichia coli* (STEC) strains detection [48]. In terms of viable bacterial cell, developed LAMP assay exhibited a 10-fold lower detection limit than conventional PCR, detecting as low as 81 CFU/mL of EAEC in pure culture, compared to 8.1 × 10^2^ CFU/mL by PCR. Furthermore, in spiked stool samples, LAMP detected 8.1 × 10² CFU/g, whereas PCR required 8.1 × 10⁴ CFU/g. The difference in sensitivity between fecal sample and pure culture maybe related to the presence of inhibitors, such as bile salts and plant-based polysaccharides commonly found in fecal samples [49]. A study on patients with diarrhea reported a bacterial count in diarrheal samples around 2.1 × 10⁹ CFU/g, versus healthy controls averaging ~ 5.6 × 10¹⁰ CFU**/**g [50].

Among diarrheal cases where EAEC was the pathogen detected, the bacterial load was around 2.78 × 10⁶ bacteria/g [51]. In this study, the developed LAMP assay enables sensitive detection of EAEC in stool samples, with a minimum detectable load of 8 × 10² CFU/g under controlled experimental conditions. Clinical stool specimens were not included in this study due to limited availability. Therefore, all experiments were conducted using spiked stool samples to evaluate the analytical performance of the LAMP assay under controlled reaction conditions and showed high analytical sensitivity and specificity, further clinical validation by using patient samples should be conducted to assess the assays overall performance. Regarding cost and time, LAMP offer a significant advantage over conventional PCR. LAMP operates under isothermal conditions to produce the results within 60 min. This is considerably faster than the 1.5 to 3 h required for PCR assays. In terms of cost LAMP is more cost effective than PCR. The minimal equipment and simplified protocols make LAMP especially suitable for use in low-resource clinical settings [52].

## Conclusion

The developed LAMP assay has a capability to be alternative detection methods to address the shortage of efficient diagnostic tools for EAEC. While the developed assay reliably detects *aaic*-positive strains, it may not detect EAEC strains lacking this gene, yet it offers a potential basis for the development of multiplex LAMP assays for comprehensive EAEC detection. The developed assay demonstrated high analytical sensitivity and specificity in limited strain panel and has potential as an alternative to PCR, pending further clinical validation using patient sample.

## Data Availability

All data generated and analyzed in this study are available within the manuscript.

## Abbreviations

EAEC: Enteroaggregative Escherichia coli
HEp-2: human epithelial cell
TP: True positive
TN: True negative
NPV: Negative predictive value
PPV: positive predictive value
NC: Negative control
